# Strain Wave Acquisition by a Fiber Optic Coherent Sensor for Impact Monitoring

**DOI:** 10.3390/ma10070794

**Published:** 2017-07-13

**Authors:** Claudio Sbarufatti, Alessio Beligni, Andrea Gilioli, Maddalena Ferrario, Marco Mattarei, Mario Martinelli, Marco Giglio

**Affiliations:** 1 Dipartimento di Meccanica, Politecnico di Milano, Milano 20156, Italy; alessio.beligni@polimi.it (A.B.); andrea.gilioli@polimi.it (A.G.); marco.giglio@polimi.it (M.G.); 2 Dipartimento di Elettronica, Informazione e Bioingegneria, Politecnico di Milano, Milano 20133, Italy; maddalena.ferrario@polimi.it (M.F.); marco.mattarei@polimi.it (M.M.); mario.martinelli@polimi.it (M.M.)

**Keywords:** Lamb wave, coherent detection, interferometric fiber optic sensors, impact force reconstruction, finite element model, modelling

## Abstract

A novel fiber optic sensing technology for high frequency dynamics detection is proposed in this paper, specifically tailored for structural health monitoring applications based on strain wave analysis, for both passive impact identification and active Lamb wave monitoring. The sensing solution relies on a fiber optic-based interferometric architecture associated to an innovative coherent detection scheme, which retrieves in a completely passive way the high-frequency phase information of the received optical signal. The sensing fiber can be arranged into different layouts, depending on the requirement of the specific application, in order to enhance the sensor sensitivity while still ensuring a limited gauge length if punctual measures are required. For active Lamb wave monitoring, this results in a sensing fiber arranged in multiple loops glued on an aluminum thin panel in order to increase the phase signal only in correspondence to the sensing points of interest. Instead, for passive impact identification, the required sensitivity is guaranteed by simply exploiting a longer gauge length glued to the structure. The fiber optic coherent (FOC) sensor is exploited to detect the strain waves emitted by a piezoelectric transducer placed on the aluminum panel or generated by an impulse hammer, respectively. The FOC sensor measurements have been compared with both a numerical model based on Finite Elements and traditional piezoelectric sensors, confirming a good agreement between experimental and simulated results for both active and passive impact monitoring scenarios.

## 1. Introduction

Structural Health Monitoring (SHM) as a technology offers the promise of reduced maintenance costs and increased operation safety for a wide range of engineering structures, including mechanical, aerospace and civil application. In general, SHM combines sensors, signal processing, feature extraction techniques, and modelling strategies in order to perform the automated diagnosis of structural components. This includes, on one hand, load monitoring, that identifies extreme load scenarios like those related to impacts, and on the other hand, damage identification, e.g., due to fatigue ageing or corrosion [1,2,3,4,5], that comprises anomaly detection, damage type evaluation, localization, quantification, and the final prognosis of its future evolution.

Focusing on impact monitoring, two complementary approaches are mainly adopted in literature for impact detection and, subsequently, for the estimation of potential impact damage. The first method, namely passive monitoring [2,3,4,5], often referred to as impact force reconstruction [6], identifies an impact by analyzing the time-series strain data generated by the impact itself, which provide information about impact occurrence, location, and force. The second approach, namely active monitoring [1], can be performed by generating travelling waves, typically by means of piezoelectric transducers, which propagate inside a specimen at ultrasonic speed and are then detected by a distributed sensor network. Several features can be identified in the acquired signals, which can be directly related to specific structural impact damages that occurred along the wave path. If bounded media with relatively small thickness are concerned, like in most of the cases reported in literature [3] and in the present work, this method is often referred to as Lamb wave monitoring [7,8,9].

Both passive and active monitoring strategies involve the acquisition of dynamic signals generated either by the occurrence of an impact or by an active transducer, thus requiring the sensor network to be associated with high frequency acquisition systems. This has been done so far by means of strain gauges and accelerometers [10], typically exploiting a network of piezoelectric transducers connected to high sampling rate oscilloscopes (MS/s) [11]. More recently, optical fiber sensors (OFSs) have been also proposed for impact monitoring [12,13], impact force reconstruction [6], and Lamb wave monitoring [14] due to their greater sensitivity and reliability, reduced size and weight, cost-effectiveness, and immunity to electromagnetic interference, which make them attractive especially for those applications where safety represents a critical issue. However, most of the works reported in literature have been performed by means of Fiber Bragg Gratings (FBGs) [3,12], which ensure good reliability and high signal-to-noise ratios. Nevertheless, their acquisition systems become all the more demanding as the frequencies of the monitored phenomena increase. Monitoring of mechanical wave propagation inside composite materials has been also recently proved by exploiting dynamic strain Brillouin sensors [15,16], which can provide appealing strain distributed information along the entire sensing fiber, yet requiring a complex and expensive interrogation HW. In this framework, optical fiber interferometers represent a valid alternative for acoustic and ultra-acoustic sensing due to their extensively proved, highly-sensitive monitoring capabilities with bandwidth up to MHz [12], resulting in an interesting compromise every time a specific punctual SHM applications requires HW simplicity and reduced costs.

The interferometric approach is adopted in this study to acquire dynamic signals for active and passive impact monitoring in metallic structures. In particular, it exploits a novel sensing solution based on standard optical fibers associated to an innovative phase-diversity coherent detection scheme, which retrieves in a completely passive way the high-frequency phase information carried by the optical signal. The sensing fiber can be configured in different layouts in order to meet the requirements of the specific monitoring application, finding the proper tradeoff between the required sensitivity and need for a local measure. As an example, as suggested by several works related to ultrasonic wave detection with FBG [17,18,19], an accurate detection of high frequency signals reasonably requires a relatively short gauge length, which, in turn, can limit the sensitivity of the sensor itself. In this work, the sensing fiber is arranged into multiple loops glued only for a short length to the structure in order to increase the interferometric sensitivity while still guaranteeing a localized measurement, as required for the acquisition of very high frequency dynamics. A first experimental proof of this solution, which combines sensing fiber coils with an interferometric approach has been recently demonstrated in [20,21] for both passive monitoring of acoustic emission and active damage monitoring based on ultrasonic wave scattering. In this work, for the first time, we describe in detail how the sensor configuration, intended as the gauge length and the number of the optical fiber loops, can been selected to maximize the performances of the coherent fiber optic monitoring solution for two highly dynamic cases, namely Lamb and impact strain wave signals, for active and passive impact monitoring applications. Specifically, the focus of this work is the analysis of the quality of the acquired sensor signal in different layouts and not the inference on either the impact or the impact damage features. The strain waves generated first by a piezoelectric transducer and then by an impulse dynamometric hammer on an aluminum skin panel are acquired by the fiber optic coherent (FOC) sensor and then signals are verified by comparison with a numerical Finite Element Model (FEM) and with traditional piezoelectric (PZT) sensors.

A detailed introduction to the sensing principle is presented in Section 2, followed by a description of the experimental setup in Section 3. Section 4 provides information on the adopted Finite Element (FE) modelling strategy. Results for active monitoring and passive impact identification are shown in Section 5 and Section 6, respectively. A conclusive section is finally provided.

## 2. Sensing Principle

**Figure 1:**
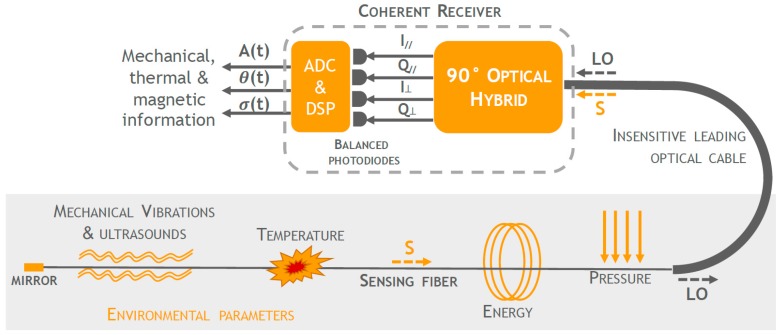
Polarization and phase diversity coherent detection scheme exploiting a 90° optical hybrid.

The interferometric fiber optic sensing solution exploited in this work for ultrasonic strain wave detection relies on a novel and recently patented phase-diversity coherent detection scheme [22]. The proposed approach exploits a standard, disposable optical fiber as the sensing element capable to monitor different parameters related to health conditions of mechanical, civil, and aerospace structural systems, such as temperature, deformations, vibrations, ultrasounds, and even presence of electromagnetic fields, thus replacing the need of a plurality of conventional sensors. All these parameters induce fiber elongations and refractive index changes, which, in turn, result in variations of the amplitude *A*(*t*), phase *θ*(*t*), and polarization *σ*(*t*) of the optical field S that propagates in the fiber. Differently from conventional interferometric approaches, the key element of the proposed approach is represented by a new polarization and phase diversity coherent demodulation scheme (Figure 1) where the signal S, back-reflected from the sensing fiber end, and a local oscillator (LO) are combined in a 90° optical hybrid [23,24,25], enabling the extraction of phase, amplitude, and polarization of the optical field S. Indeed, the outputs of the 90° optical hybrid, used in combination with balanced photodetectors, provide the in-phase *I* and the quadrature *Q* signals for both polarization *σ*(*t*) components (// and ⏊) of S, where I÷S·LO·cos(θ(t)) and Q÷S·LO·sin(θ(t)). The four in-phase *I* and quadrature *Q* signals are then digitalized and post-processed with demodulation algorithms exploited in coherent optical communications [26,27], allowing the recovery of *A*(*t*), *θ*(*t*), and *σ*(*t*) in a completely passive way without the need of complex active feedbacks for quadrature point stabilization as in conventional fiber optic interferometric and polarimetric schemes [28,29]. Moreover, in the configuration of Figure 1, the signal S and the LO follow the same path inside a leading cable until the area to be monitored so that the useful information results exclusively that integrated along the sensing fiber length and all environmental perturbations acting on the leading cable result in a common-mode phase noise which is not detected at the receiver.

**Figure 2:**
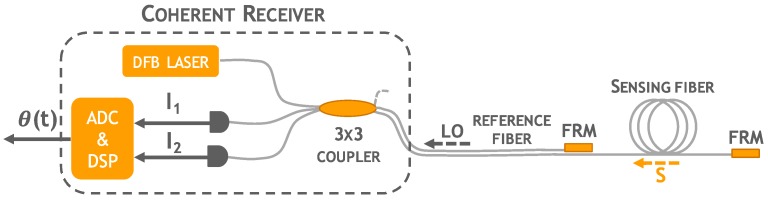
Phase-diversity coherent detection scheme exploiting a 3 × 3 optical coupler. The third 3 × 3 output is properly terminated to avoid light reflections.

As mentioned, the proposed approach allows even magnetic fields measurements (i.e., current or voltage measurements), carried out by analyzing the evolution of the polarization *σ*(*t*) of the back-reflected signal S. However, when the monitoring of *σ*(*t*) is of no interest, as in the present application, the 90° optical hybrid of Figure 1 can be replaced by a simpler phase-diversity coherent receiver recovering just phase *θ*(*t*) and amplitude *A*(*t*) information of S. In this case the coherent receiver comprises only a 3 × 3 optical coupler (Figure 2) where two of the three output fibers, constituting the sensing and reference arms of the interferometer, are both terminated with Faraday Rotator Mirrors (FRM) which retrace the S and LO polarizations [30] and always guarantee a mutually parallel fixed polarization state at the 3 × 3 coupler, thus ensuring maximum visibility of the signals I and Q. In this way, *θ*(*t*) and *A*(*t*) can be properly reconstructed regardless of random signal polarization fluctuations that inevitably occurs along the fiber and which may cause signal fading in conventional interferometric detection schemes [31].

A standard telecommunication distributed feedback laser (DFB) at 1550 nm and spectral linewidth of Δ*ν* = 10 MHz, corresponding to a coherence length *L_c_* of approximately 6 m, is coupled to one input of the 3 × 3 coupler and the optical signal split through both reference and sensing arms. The back-reflected optical signals S and LO are then collected at the two other input ports of the 3 × 3 coupler and detected by a pair of photoreceivers which provide the following normalized output signals [32]:

(1) $$I_{1}=C+Bcos\left(\theta \left(t\right)-\frac{2}{3}\pi \right),\;I_{2}=C+Bcos\left(\theta \left(t\right)+\frac{2}{3}\pi \right),$$

In (1), *B* and *C* are constants that depend on the 3 × 3 coupler and *θ*(*t*) is the phase difference induced by strain variation Δ*L* sensed by the sensing arm according to the following relation:

(2) $$\theta \left(t\right)=\frac{2\pi }{\lambda }n2∆L\left\{1-\frac{n^{2}}{2}\left[P_{12}-\nu \left(P_{12}+P_{11}\right)\right]\right\},$$

where *λ* is the optical wavelength, *n* refractive index, *ν* the Poisson’s ratio, and *P_ij_* the strain-optic tensor of the optical fiber. Compared with the coherent receiver of Figure 1 the 3 × 3 coupler provides two output signals *I*_1_ and *I*_2_ displaced by 120° and not by 90°. Yet, several demodulation methods can be exploited [32] which yield the in-phase *I* and the quadrature *Q* components of the optical signal S, from which, by calculating the arc-tangent of the ratio between *I* and *Q*, the phase difference *θ*(*t*) is retrieved.

In the present work, the phase-diversity coherent approach is exploited to retrieve high-frequency information induced in the phase *θ*(*t*) of the optical signal by the propagation of ultrasonic Lamb waves in metallic structures. Indeed, the proposed sensing solution offers the possibility of a real-time broadband spectral analysis, mainly limited by the acquisition bandwidths (MSamples/s) of the Analog to Digital Converter (ADC) and the Digital Signal Processing (DSP), and is therefore suitable to monitor and detect even fast transients and ultrasounds. Furthermore, the high linearity and sensitivity of coherent detection [33] guarantees sensitivity in phase measurements down to μrad which, combined with an optimized choice of the sensing fiber length and its arrangements, can result in strain resolutions in the order of με with wide dynamic ranges of ±10^4^ με.

## 3. Materials and Methods

One main advantage of the FOC sensor, e.g., compared to Fiber Bragg Gratings (FBG), is the possibility to be tailored for the specific application. Focusing on high frequency dynamics, several works related to FBG acquisition have shown that a key parameter affecting the grating response is the ratio between the ultrasonic wavelength and the grating length [17,18,19]. The same considerations reasonably hold for interferometric sensors, which perform a spatial integration of the ultrasonic signals over the entire sensing length: if an excessive sensing length is chosen, the overall integration may drastically degrade the sensor sensitivity. A compromise must be found by increasing the gauge length to provide sufficient sensitivity without compromising the reliability of the measure. A description of the method used to select the best FOC sensor layout is presented in the following, specifically for the active and passive monitoring cases.

### 3.1. Experimental Setup

**Figure 3:**
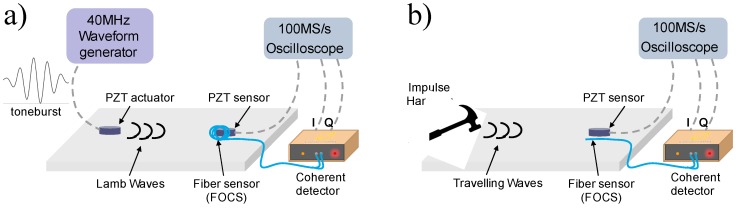
Test layout for (**a**) active and (**b**) passive impact monitoring cases. FOCS: fiber optic coherent sensor. PZT: piezoelectric.

**Figure 4:**
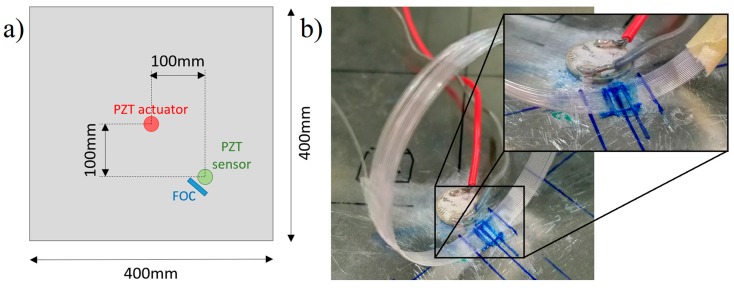
(**a**) Schematic of the actuator and sensor layout for active monitoring tests; (**b**) detail of the sensing optical fiber arranged in 20 loops and glued to the aluminium plate for active monitoring tests.

The experimental setups are shown in Figure 3 and a schematization of the specimen and relative transducer positions is also presented in Figure 4a. A square aluminium skin plate is considered to investigate the effect of different FOCS layouts on the quality of the acquired dynamic signals. The 400 × 400 mm skin plate is made of aluminium Al2024 with a thickness of 1.5 mm. The optical fibers exploited with the proposed coherent sensing technology are standard G.652 single-mode optical fiber with 9 μm mode field diameter, a 125 μm cladding, and a 242 μm acrylate coating. Two PZT transducers (PIC-255) with 10 mm diameter and 1 mm thickness have been placed as shown in Figure 4a and used, respectively, as actuator and sensor. The PZT actuator was used for active monitoring only, modulated by a waveform generator (TTi TGA1242-40MHz bandwidth). The PZT sensor and the FOC signals were acquired by means of a 15-bit Teledyne LeCroy’s HDO6104 oscilloscope at 100 MS/s. The PZT sensor, in particular, was located in correspondence of the FOC sensor position, for a proper comparison of both acquired signals. For passive monitoring, the central PZT actuator is not used and it is substituted by a dynamometric hammer excitation. In particular, the impulse hammer adopted is a PCB 086C03, with a teflon tip (PCB 084B04), representing a medium stiffness material. The same positions are kept for the PZT and the FOC sensors during active and passive monitoring cases. 

### 3.2. FOC Sensor Configuration for Active Monitoring

Active impact monitoring consists in the exploitation and analysis of Lamb wave scattering for impact damage identification. However, the main goal of this activity is to evaluate the quality and features of the FOC sensor signal for active monitoring applications based on Lamb wave pitch-catch mode, and thus no damages are considered at this stage. The Lamb waves are generated by driving the PZT actuator with a 5-cycle toneburst, shaped with a Hamming window. Different frequencies have been selected, ranging from 50 kHz to 200 kHz, to verify the FOCS signal in comparison with a numerical model and signals acquired by the PZT sensor.

It is known that group and phase velocities of wave propagation modes depend on the thickness–frequency product, based on dispersion curves [8,9]. Group and phase velocities values have been calculated with *Vallen Dispersion R2014* according to the parameters of the skin plate material, e.g., Young modulus E=68,710 MPa, Poisson ratio ν=0.33 and density $\rho =2.78\;\mathrm{Kg}/{\mathrm{dm}}^{3}$ and reported in Table 1 as a function of the excitation frequency (*f*) for the S0 and the A0 fundamental propagation mode only [8,9]. The calculated theoretical group velocities (*c_g_*) have been used to predict and verify the Time of Arrival (ToA) of the experimental wave packets, whereas the phase velocities (*c_p_*) have been used to calculate the spatial wavelength (*λ*) of the travelling wave, according to the relation λ=cpf. Looking at the *λ* values in Table 1, it is clear that the A0 requires a more local measure for its correct identification, since it is associated with a smaller wavelength. Based on this preliminary consideration, a gauge length ranging from 4 mm to 16 mm has been selected and tested during active monitoring tests.

Furthermore, in order to increase the overall sensing length and minimize the local integration length, the sensing fiber was arranged in several loops and only few mm/loops were attached to the aluminium skin panel, as shown in Figure 4b. Experimental tests have been performed with several loop arrangements, varying the number of loops and the fiber gauge length, in order to define, in dependence of the frequency range to be monitored, the optimal configuration in terms of sensitivity and quality of detected ultrasonic strain waves. 

### 3.3. FOC Sensor Configuration for Passive Monitoring

**Figure 5:**
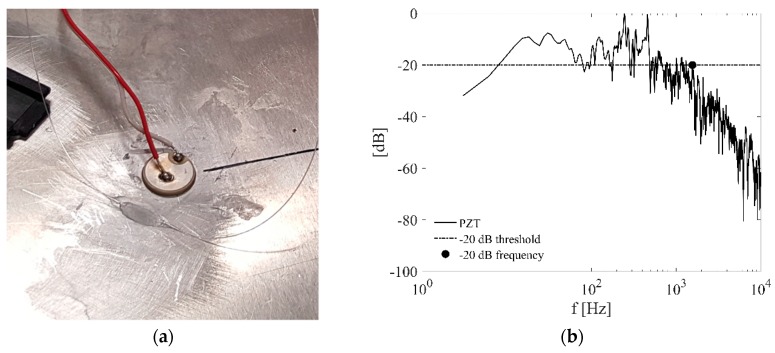
(**a**) Detail of the sensing optical fiber glued to the aluminium plate for passive impact monitoring; (**b**) Power spectral density of the impact response of the PZT sensor located in the vicinity of the FOC sensor.

The FOC sensor configuration selected for active monitoring tests is not properly suitable to perform passive monitoring characterizations. In fact, the occurrence of an impact close to the sensor generates mechanical vibrations of the sensor loops, thus altering the acquired signal and preventing the extraction of the actual impact features, i.e., the ToA and the signal peaks, required for impact localization and energy estimation, respectively. One possibility, as described in [20], is to directly bond the entire coil to the structure surface, however this means losing control of the strain direction and affecting the comparison of the strain signal with the prediction by the numerical Finite Element model presented in Section 4. The multiple-loop configuration is thus replaced in this case by a linear layout of the sensing fiber on the metallic structure, as shown in Figure 5a, where the sensor sensitivity is improved by just increasing the fiber gauge length. Indeed, if the Welch’s power spectral density of the PZT signal during an impact with dynamometric hammer is taken for reference (Figure 5b), it is clear that the main frequency content will be found up to 1563 Hz, if the −20 dB threshold is considered, thus taking the 99.99% of the signal power into account. In this range of frequencies, the phase velocity can be evaluated through the flexural wave formulation, with 149.65 m/s corresponding to 1563 Hz. This implies a spatial wavelength equal to 95.7 mm. A 20 mm gauge length has thus been selected resulting in a good signal sensitivity and also allowing further margin of improvements as the selected gauge length is still far below the spatial wavelength associated to the main frequency content of the impact. Figure 5a shows the FOC sensor configuration for passive impact monitoring with a PZT placed next to the sensing fiber for comparison.

## 4. Finite Element model

**Figure 6:**
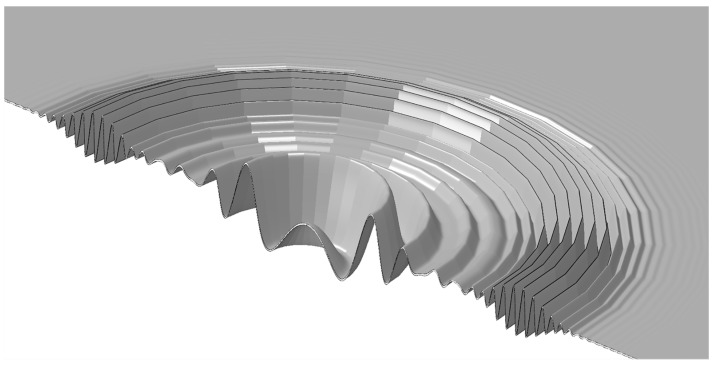
Example of tone burst displacements as a result of the axis-symmetric model.

**Figure 7:**
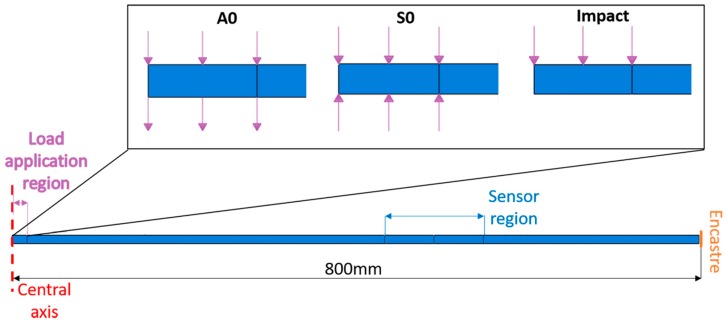
Schematic of the axis-symmetric Finite Element model (FEM) of the plate, with indication of the three different load configurations selected to model A0 and S0 Lamb wave propagation modes and the impact.

A numerical model is used in this work to verify the features of the signals provided by the FOC sensor. An axis-symmetric Finite Element (FE) model has been developed in order to numerically reproduce the strain wave propagation caused by both a toneburst (Figure 6) and a real impact, for active and passive impact monitoring respectively. An axis-symmetric structure model, reasonable for this specific scenario, has been selected to reduce computation costs. It is worth mentioning that axis-symmetric modelling can be exploited only in cases when both the geometry and the load are axis-symmetric. In the present case, the load is central but the real specimen geometry is square. However, in this work the numerical model is used to verify the portion of the signal that is relative to the arrival of the first wave packets at the sensor location. This specific part of the signal is not affected by the size and the boundaries of the target as long as the sensor location is enough far away from the boundaries to neglect the effect of wave reflections. In practice, the FE model is a disk with an 800 mm radius, while the real structure is a 400 mm × 400 mm square plate. The position of the sensors in the numerical model is selected in order to reproduce the experimental distance between the load application point and measurement distances. In Figure 7, the FE model is reported without the mesh. It shows the central rotation axis, the area where the load is applied, and the location of the sensor.

As reported in Figure 7, a clamp boundary condition is applied on the external side of the target plate, thus fixing all the degrees of freedom. As mentioned before, the numerical plate size is properly selected so that the signal associated to the first fundamental mode wave passing through the sensor is not affected by boundary conditions, thus allowing a direct comparison with the experimental signals. The input of the model is a load vs. time curve representing a tone-burst with a certain frequency or the load vs. time curve measured by the load cell of the dynamometric hammer, to emulate both, respectively, active and passive monitoring cases. Such load is applied to the plate in terms of a pressure acting on an area corresponding to either the PZT actuator area or the contact area of the dynamometric experimental tip, for active and passive monitoring, respectively. Note that, as a model of the PZT actuator is not included into the analysis, the A0 and S0 modes have been separately considered in different simulation models, specifically providing a pressure excitation as schematized in Figure 7. The output of the model is the strain measured on the top surface in the sensor area. In particular, the output strain signal is proportional to the integral of the strain in the sensor region, whose dimension changes as a function of the exploited fiber optic gauge length.

The FE model has been built using the commercial software ABAQUS 2016 and its explicit solver. The explicit solver is recommended for very non-linear applications or, like in this case, for reproducing highly dynamic problems. Reduced axisymmetric CAXAR elements have been adopted having a size of 0.1 × 0.1 mm, thus including 15 elements along the plate thickness. In total, the entire model includes 75,000 elements. The calculation time with an Intel Xeon 16 CPUs at 2.4 Ghz workstation is approximately one hour.

## 5. Results for Active Lamb Wave Monitoring

In this section FOC sensor performances in terms of sensitivity are evaluated for the active monitoring case, as a function of both the number of optical fiber loops (Section 5.1) and the gauge length (Section 5.2), that is the length of the fiber actually attached to the monitored specimen. The FOC sensor behavior in response to increasing tone-burst frequencies of the PZT actuator is finally presented in Section 5.3 for a specific sensor configuration. Both the analytical model, based on dispersion curves, and the numerical model, based on Finite Element simulations, are used as a term of comparison to verify the acquired experimental signals. Specifically, the analytical model is used to predict the arrival times of the wave packets associated to the fundamental modes while the numerical model provides a better understanding of the detected signal shape.

### 5.1. Effect of a Different Number of Optical Fiber Loops

The effect of a different number of optical fiber loops on the FOC sensor signal is investigated in the range between 5 and 20 loops. The lower limit is imposed by the required minimum sensitivity, while the upper bound is provided by practical difficulties in the manual arrangement of loops. In particular, the minimum strain sensitivity was limited in this experiment to only few mε due to the 15 resolution bits of the high-bandwidth oscilloscope, but sensitivity down to με, as those of FBG, can be achieved with higher performance ADC acquisition boards. Yet, one great advantage of coherent (interferometric) sensors, with respect to FBG, is that their sensitivity can be increased by exploiting longer sensing length. Thus, the same FOC sensor coil has been connected and disconnected several times from the test specimen, each time adding further 5 loops. In particular, cyanoacrylate glue has been used to attach the FOC sensor coil to the aluminium plate, which facilitates the optical fiber disconnection with respect to a typical bi-component epoxy glue. The length of the glued coil section was fixed to approximately 4 mm and the toneburst frequency was driven at 50 kHz.

**Figure 8:**
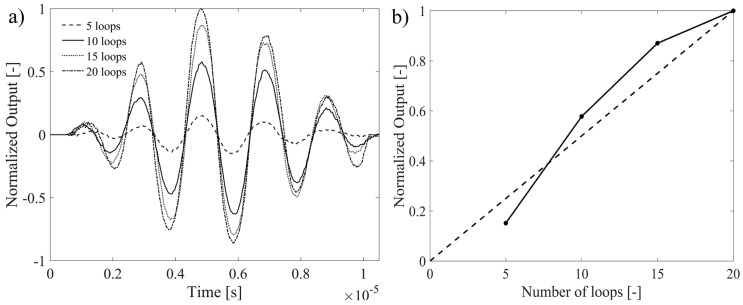
Toneburst signals acquired by the FOC sensor as a function of different number of fiber loops.

The acquired toneburst signals are reported in Figure 8, focusing on the time window associated with the first A0 wave packet. Signals have been normalized with respect to the maximum peak measured by the 20-loops configuration. Considering that the FOC sensor response corresponds to the integral of the strains over the entire sensing fiber length, a linear magnification of the signal peaks is expected as a function of the loop number. In fact, this is true when looking at signals relative to 10 and 15 loops, while a slight departure from linearity is found for the remaining cases. This is due to several factors influencing the strain transfer from the aluminium plate to the FOCS, such as (i) the thickness of the bonding layer, as indicated in [34]; (ii) the stiffness of the bonding layer itself and (iii) the repeatability of the actual gauge length in sequential sensor installations.

### 5.2. Effect of Different Gauge Lengths

The effect of different gauge lengths on the acquired FOC sensor signal is investigated in this section with a two-fold objective: (i) understanding the effect of increasing spatial integrations on the detected FOC sensor signal quality, in comparison with a numerical model and (ii) defining a threshold ratio $L_{g}/\lambda$, below which a reliable measure of the ultrasonic wave dynamics is guaranteed. This has been carried out specifically for the A0 wave propagation mode, as it has been identified as the most critical in Section 3.2. The toneburst frequency was set to 50 kHz and the ratio $L_{g}/\lambda$ was gradually increased from 0.25 (corresponding to $L_{g}=4\;\mathrm{mm}$) to approximately 1 (corresponding to $L_{g}=16\;\mathrm{mm}$).

**Figure 9:**
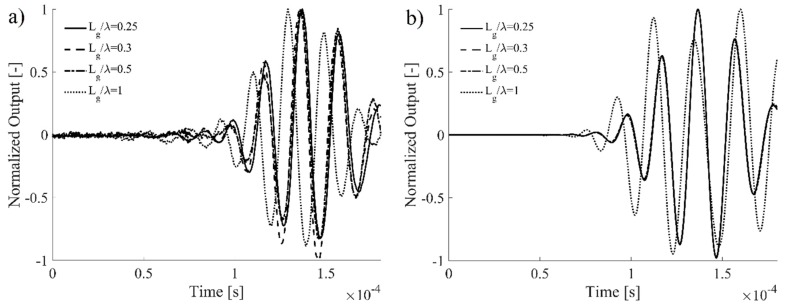
Gauge length effect on the Lamb wave signal acquired by the FOC sensor: (**a**) experimental results and (**b**) numerical simulation. Results are presented as a function of $L_{g}/\lambda$.

The time signals acquired by the FOC sensors are shown in Figure 9a and compared with the numerically simulated signals in Figure 9b. For a proper comparison, signal amplitudes have been normalized in order to obtain a unitary maximum strain peak. Looking at the A0 first wave packet, a very high correlation was experimentally found among the acquired FOCS signals characterized by $L_{g}/\lambda$ less than 0.5. This is confirmed by the FEM analysis, which provides perfectly superposed signals when $L_{g}/\lambda$ is less than 0.5. In proximity of a unitary ratio of $L_{g}/\lambda$, the signal manifests an apparent phase shift, caused by the spatial integration of a non-axis-symmetric wave packet. Moreover, a good agreement between experimental and numerical results is found at small $L_{g}/\lambda$ in terms of signal shape for different gauge lengths.

### 5.3. Effect of Increasing Toneburst Frequency

**Figure 10:**
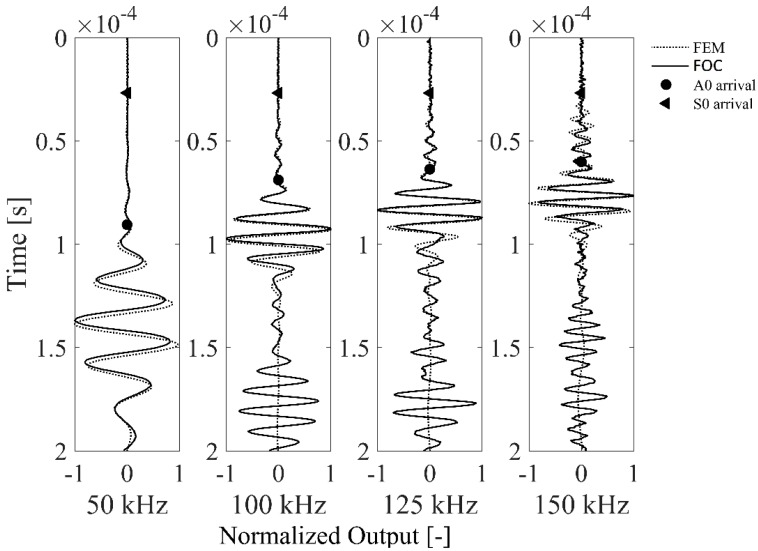
Comparison of FOC sensor vs. FEM simulated signals at increasing Lamb wave frequencies. The analytical time of arrival (ToA) for A0 and S0 modes is also provided as reference.

The FOC sensor signal behavior for increasing toneburst frequencies is now investigated in comparison with the numerically simulated results. Figure 10 shows the FOC sensor and the FE signals relative to four different toneburst frequencies, namely 50 kHz, 100 kHz, 125 kHz, and 150 kHz. The FOC sensor was arranged in 20 optical fiber loops and the gauge length (*L_g_*) was fixed to 2 mm, meaning a different ratio $L_{g}/\lambda$ at each investigated frequency, respectively 0.12, 0.18, 0.20, and 0.22, according to the phase velocity of the A0 wave propagation mode reported in Table 1. The foreseen analytical ToA for A0 and S0 modes is also reported as reference in Figure 10. Several considerations can be set out.

First, a very high correlation between the FOC sensor signals and numerical prediction is found up to 150 kHz; this proves the validity of the axis-symmetric modeling approach and allows a correct interpretation of the experimental signal components. It is known from previous studies reported in the literature that different sensitivities to fundamental strain wave modes exist as a function of the toneburst frequency [35]. Thus, two separate simulation models have been run at each frequency for the A0 and S0 fundamental modes, respectively, and the resulting simulated time-series have been linearly superposed to fit the experimental signals. Accordingly, the FOC sensor signal manifests a predominant sensitivity to the A0 mode at lower frequencies, that becomes comparable to the S0 sensitivity at higher frequencies. It must be remind that the FEM simulation do not account for boundary reflections as a wider circular plate with 800 mm radius has been chosen purposely.

**Figure 11:**
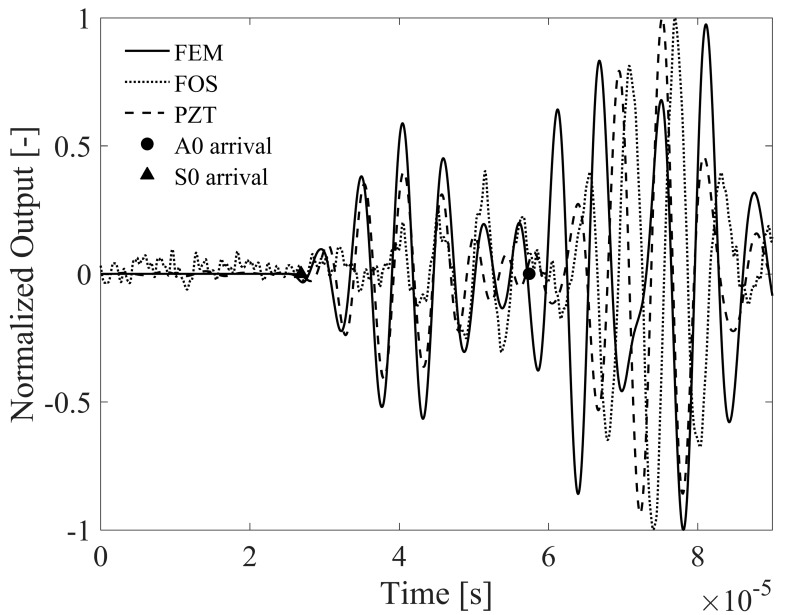
Comparison of FOC and PZT sensors vs. FEM simulated signals at 175 kHz Lamb wave frequency. The analytical ToA for A0 and S0 modes is also reported as reference.

Second, a lower sensitivity of this FOC sensor configuration to the S0 mode is found, specifically at higher frequencies, which hampers an exhaustive signal comparison with the FEM. This is confirmed in Figure 11, where the normalized FOC sensor signal is shown along with the numerical simulation and the signal simultaneously measured by a PZT sensor in presence of a 175 kHz toneburst. The FOC sensor sensitivity is reduced not due to a limit in its bandwidth, neither due to an excessive gauge length, but because of the limited strain transfer capability of the bonding layer and in turn of the optical fiber coating. The measured PZT signal instead indicates that the FE model correctly predicts the signal behavior also at higher frequencies.

Finally, referring to Table 1, it is clear that the S0 mode is characterized by larger spatial wavelengths, thus allowing for a longer gauge length compared with A0. In fact, it was proven experimentally (though not reported here) that if a bigger sensitivity to S0 is required, a larger gauge length needs to be selected, although hampering the A0 mode acquisition.

## 6. Results for Passive Impact Monitoring

**Figure 12:**
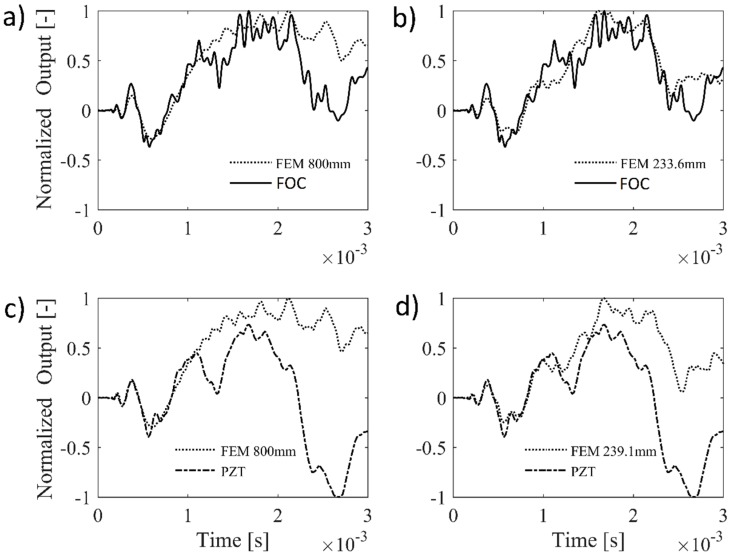
Comparison of FOC (**a**,**b**) and PZT (**c**,**d**) sensors vs. FEM simulated signals after an impact event. On the left (**a**,**c**), no strain wave reflection is included in the FEM approximation. On the right (**b**,**d**), FEM is designed to account for first boundary reflections.

After having proved the feasibility of the FOC sensor for detection of high frequency phenomena in an active monitoring set-up, further measurements have been carried out to acquire strain travelling waves caused by an impact, as typically occurs in passive monitoring. The same test specimen as for active monitoring is considered hereafter. The FOC sensor is configured as explained in Section 3.3 and FOC signals have been compared with both a PZT sensor and the FEM results in Figure 12.

Figure 12a,c reports the FOC and PZT sensor signals, respectively, compared with numerical solutions calculated for a circular plate with 800 mm radius to exclude any boundary reflection during simulation time. It can be noted that both the FOC and the PZT sensors provide a signal that is consistent with the numerical prediction in the first instant after the impact (within 1 ms). Then, due to wave reflection and wave mode superposition, the strain field becomes more complex and the difference between the two sensor curves and the FEM increases. Furthermore, the two sensors respond in a different way to the propagating strain wave: in particular, the FOC has a preferential direction for strain measure, which in turn makes the comparison with the FEM more accurate after 1 ms.

**Figure 13:**
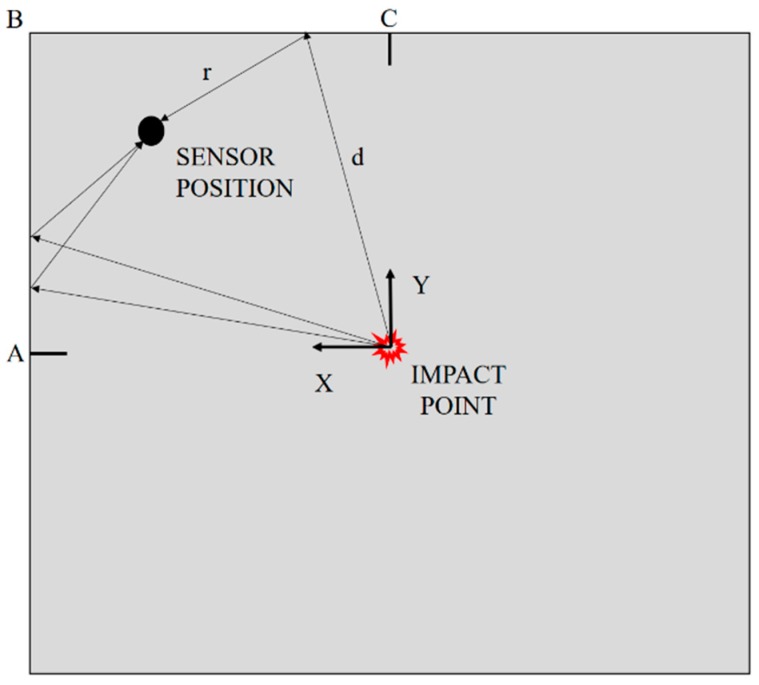
Schematic of the strain wave reflections at boundary edges after impact.

A more realistic version of the FEM has been subsequently designed in order to take the first strain wave boundary reflection into account, thus allowing a better understanding of the features of the acquired sensor signals. Referring to Figure 13, the radius of the axis-symmetric model has been selected in order to guarantee consistence with the smallest travelling path among those available for reflections in the A-B-C domain (generically defined as *d + r* in the figure). Due to a non-perfect coincidence of the FOC and PZT sensor positions, two FE models have been used in Figure 12b,d, with radius 233.6 mm and 239.1 mm for the FOC and PZT sensor signal model, respectively. This provides further improvements of numerical simulation, especially for the FOC signal.

These first results demonstrate that the FOC sensor can be a valid alternative to PZT (and FBG sensors [3]) for the extraction of useful information for passive impact identification, i.e., the identification of the ToA of the elastic waves induced by the impact.

## 7. Conclusions

A fiber optic sensing technique exploiting a novel coherent detection scheme has been proposed for both active and passive impact monitoring. The proposed approach allows different sensing fiber layouts in order to fulfil the requirements of the specific experimental measure.

For active impact monitoring relying on ultrasonic Lamb waves measurements, the sensor consists in a fiber optic coil of several loops that are bonded to the monitored specimen with a proper gauge length. The effect of loop number and gauge length on the quality of the acquired signal has been investigated, specifically for acquisition of toneburst Lamb wave signals. It has been proved that increasing the number of optical fiber loops achieves a higher sensitivity, though the improvement does not linearly scale if the bonding layer thickness is not perfectly controlled and if the strain transfer capability of the bonding layer is limited. On the contrary, it has been highlighted that an increase in the gauge length causes a larger spatial integration which, in turn, may hamper the sensor capability in detecting the actual high-frequency phenomena. In fact, on one hand a large gauge length is indeed needed to maximize sensor sensitivity, but on the other hand, a threshold integration gauge length exists strictly depending on the travelling wave speed to be measured, above which the acquired fiber optic signal is distorted.

For passive impact monitoring, the fiber coil layout causes unwanted coil oscillations during the impact, thus hampering the acquisition of the actual travelling strain wave. Yet, in this case, the sensor sensitivity could be improved by simply increasing the attached gauge length without arranging the optical fiber into multiple loops. This was possible due to the lower frequencies involved in passive impact monitoring compared with those occurring in Lamb wave active monitoring.

The feasibility of the fiber optic coherent sensor for active and passive impact monitoring has thus been demonstrated through comparison of the fiber optic sensor signals with results from both a numerical axis-symmetric Finite Element model and characterizations with conventional piezoelectric sensors. Very good agreement was found between experimental and simulated signals for both active and passive impact monitoring scenarios. This further demonstrates that FEM can be used as a valid tool to support SHM system design.

## Figures and Tables

**Table 1:** Group and phase velocity of A0 fundamental wave propagation mode and associated wavelength of the propagating wave packets, as a function of the excitation frequency *f*.

*f* [kHz]	*c_g_* (S0) [m/s]	*c_p_* (S0) [m/s]	*λ* (S0) [mm]	*c_g_* (A0) [m/s]	*c_p_* (A0) [m/s]	*λ* (A0) [mm]
50	5265	5266	105.3	1557	820	16.40
100	5261	5265	52.6	2048	1135	11.35
150	5255	5262	35.1	2346	1350	9.00
200	5246	5259	26.3	2548	1522	7.61

## References

[1] Ochôa P., Infante V., Silva J.M., Groves R.M. (2015). Detection of multiple low-energy impact damage in composite plates using Lamb wave techniques. Compos. Part B Eng..

[2] Staszewski W.J., Worden K. (2000). Impact location and quantification on a composite panel using neural networks and a genetic algorithm. Strain.

[3] Frieden J., Cugnoni J., Botsis J., Gmür T. (2012). Low energy impact damage monitoring of composites using dynamic strain signals from FBG sensors part I: Impact detection and localization. Compos. Struct..

[4] Frieden J., Cugnoni J., Botsis J., Gmür T., Coric D. (2010). High-speed internal strain measurements in composite structures under dynamic load using embedded FBG sensors. Compos. Struct..

[5] Jang B.W., Lee Y.G., Kim J.H., Kim Y.Y., Kim C.G. (2012). Real-time impact identification algorithm for composite structures using fiber Bragg grating sensors. Struct. Control Health Monit..

[6] Park C.Y., Kim J.H., Jun S.-M., Kim C.-G. (2012). Localizations and force reconstruction of low-velocity impact in a composite panel using optical fiber sensors. Adv. Compos. Mater..

[7] Worden K. (2001). Rayleigh and Lamb Waves—Basic Principles. Strain.

[8] Viktorov I.A. (1967). Rayleigh and Lamb Waves—Physical Theory and Applications.

[9] Rose J.L. (1999). Ultrasonic Waves in Solid Media.

[10] Knapp J., Altmann E., Niemann J., Werner K.-D. (1998). Measurement of shock events by means of strain gauges and accelerometers. Meas. J. Int. Meas. Confed..

[11] Di Scalea F.L., Matt H., Bartoli I. (2007). The response of rectangular piezoelectric sensors to Rayleigh and Lamb ultrasonic waves. J. Acoust. Soc. Am..

[12] Wild G., Hinckley S. (2008). Acousto-ultrasonic optical fiber sensors: Overview and state-of-the-art. IEEE Sens. J..

[13] Zhou G., Sim L.M. (2002). Damage detection and assessment in fibre-reinforced composite structures with embedded fibre optic sensors-review. Smart Mater. Struct..

[14] Betz D.C., Thursby G., Culshaw B., Staszewski W.J. (2003). Acousto-ultrasonic sensing using fiber Bragg gratings. Smart Mater. Struct..

[15] Peled Y., Motil A., Kressel I., Tur M. (2013). Monitoring the propagation of mechanical waves using an optical fiber distributed and dynamic strain sensor based on BOTDA. Opt. Express.

[16] Morosi J., Ferrario M., Boffi P., Martinelli M. Double Slope-Assisted Brillouin Optical Correlation Domain Analysis. Proceedings of the Conference on Lasers and Electro-Optics/Europe (CLEOEurope).

[17] Minardo A., Cusano A., Bernini R., Zeni L., Giordano M. (2005). Response of fiber bragg gratings to longitudinal ultrasonic waves. IEEE Trans. Ultrason. Ferroelectr. Freq. Control.

[18] Betz D.C., Thursby G., Culshaw B., Staszewski W.J. (2006). Identification of structural damage using multifunctional Bragg grating sensors: I. Theory and implementation. Smart Mater. Struct..

[19] Majewska K., Opoka S., Kudela P., Ostachowicz W. (2015). Novel FBG rosette for determining impact location in thin plate-like structure. J. Phys. Conf. Ser..

[20] Djinovic Z., Tomic M., Stojkovic M., Schmid G. Failure detection by a fiber optic low coherence interferometric sensor. Proceedings of the 5th European Workshop on Structural Health Monitoring.

[21] Djinovic Z., Stojkovic M., Tomic M. Online Structural Health Monitoring of Wire Rope by Fiber Optic Low Coherence Interferometric Sensor. Proceedings of the 6th European Workshop on Structural Health Monitoring.

[22] Martinelli M., Ferrario M. (2013). Synoptic Fiber Optic Sensor. Patent.

[23] Doerr C.R., Winzer P.J., Chen Y.K., Chandrasekhar S., Rasras M.S., Chen L., Liow T.Y., Ang K.W., Lo G.Q. (2010). Monolithic polarization and phase diversity coherent receiver in silicon. J. Lightw. Technol..

[24] Kylia–Manufacturing OEM and Custom Optical Assembly. http://kylia.com/?portfolio=90-hybrid-coh.

[25] Richter T., Kroh M., Wang J., Theurer A., Zawadzki C., Zhang Z., Keil N., Steffan A., Schubert C. Integrated Polarization-Diversity Coherent Receiver on Polymer PLC for QPSK and QAM signals. Proceedings of the Optical Fiber Communication Conference, OFC/NFOEC.

[26] Li G. (2009). Recent advances in coherent optical communication. Adv. Opt. Photonics.

[27] Tsukamoto S., Ishikawa Y., Kikuchi K. Optical homodyne receiver comprising phase and polarization diversities with digital signal processing. Proceedings of the European Conference on Optical Communications, 2006 (ECOC 2006).

[28] Jackson D.A., Priest R.G., Dandridge A., Tveten A.B. (1980). Elimination of drift in a single-mode optical fiber interferometer using a piezoelectrically stretched coiled fiber. Appl. Opt..

[29] Spammer S.J., Swart P.L. (1996). Noise Properties of a Quadrature Phase Tracker for Interferometric Optical Fiber Sensors. IEEE Trans. Instrum. Meas..

[30] Martinelli M. (1989). A universal compensator for polarization change induced by birefringence on a retracing beam. Opt. Commun..

[31] Tao P., Yan F., Ren W., Jiang W., Tan Z., Jian S. Polarization effect in the Sagnac distributed fiber-optic sensor. Proceedings of the SPIE-OSA-IEEE Asia Communications and Photonics.

[32] Boffi P., Cattaneo G., Amoriello L., Barberis A., Bucca G., Bocciolone M., Collina A., Martinelli M. (2009). Optical fiber sensors to measure collector performance in the pantograph-catenary interaction. IEEE Sens. J..

[33] Udd E. (1991). Fiber Optic Sensors: An Introduction for Engineers and Scientists.

[34] Zhou G.-D., Li H.N., Ren L., Li D.S. Influencing parameters analysis of strain transfer in optic fiber Bragg grating sensors. Proceedings of the SPIE—The International Society for Optical Engineering.

[35] Staszewski W.J., Lee B.C., Mallet L., Scarpa F. (2004). Structural health monitoring using scanning laser vibrometry: I. Lamb wave sensing. Smart Mater. Struct..

